# Efficacy of Synbiotics for Treatment of Bacillary Dysentery in Children: A Double-Blind, Randomized, Placebo-Controlled Study

**DOI:** 10.1155/2016/3194010

**Published:** 2016-11-30

**Authors:** Manijeh Kahbazi, Marzieh Ebrahimi, Nader Zarinfar, Mohammad Arjomandzadegan, Taha Fereydouni, Fatemeh Karimi, Amir Reza Najmi

**Affiliations:** Infectious Diseases Research Centre (IDRC), Arak University of Medical Sciences, Arak, Iran

## Abstract

Bacillary dysentery is a major cause of children's admission to hospitals. To assess the probiotic and prebiotic (synbiotics) effects in children with dysentery in a randomized clinical trial, 200 children with dysentery were studied in 2 groups: the synbiotic group received 1 tablet/day of synbiotic for 3–5 days and the placebo group received placebo tablets (identical tablet form like probiotics). The standard treatment was administered for all patients. Duration of hospitalization, dysentery, fever, and the weight loss were assessed in each group. It was concluded that there was no significant difference in both groups in the baseline characteristics. The mean duration of dysentery reduced (*P* < 0.05). The mean duration of fever has been significantly reduced in the synbiotic group (1.64 ± 0.87 days) in comparison to the placebo group (2.13 ± 0.94 days) (*P* < 0.001). Average amount of weight loss was significantly lower in the synbiotic group in comparison to that in the placebo group (129.5 ± 23.388 grams and 278 ± 28.385 grams, resp.; *P* < 0.001). There was no significant difference in the mean duration of hospitalization in both groups (*P* > 0.05). The use of synbiotics as an adjuvant therapy to the standard treatment of dysentery significantly reduces the duration of dysentery, fever, and rate of weight losses. The trial is registered with IRCT201109267647N1.

## 1. Introduction

Bacillary dysentery is a disease in the category of acute infectious diarrhea. This is mostly spread by the following Gram-negative bacteria:* Shigella flexneri*,* S. dysenteriae*,* S. boydii*, and* S. sonnei* [1]. Shigella is a pathogen transmitted through the fecal-oral route, primarily via person-to-person contact. Shigellosis in children has variable symptoms ranging from a mild, self-limited diarrhea without inflammation to a severe, inflammatory, bloody diarrhea with high fever, abdominal cramps, vomiting, lack of appetite, toxic appearance, painful defecation, and other extraintestinal complications [2]. Shigellosis is estimated to be responsible for about 170 million cases and 14,000 deaths worldwide annually and such a burden is a major health problem with socioeconomic consequences [3]. Estimation of the disease inflictions remains largely speculative because only a small percentage of patients seek medical treatments and are diagnosed through stool cultures [4]. The most common microorganisms diagnosed in developing countries are* S. flexneri* and* S. dysenteriae*, but* S. sonnei* frequently causes community-wide outbreaks in industrialized countries [5]. The widely accepted definition of probiotics is as follows: “the live microorganisms which when administered in adequate amounts confer a health benefit on the host” [6]. Probiotics are mostly species of the* Lactobacillus*,* Bifidobacterium*, and* Streptococcus* genera. Also, in some studies, yeasts, such as* Saccharomyces boulardii*, have also been suggested and are used as probiotics [7–9]. In some in vitro studies,* Lactobacillus acidophilus* has been effective against some intestinal pathogen elements such as* Shigella*,* Salmonella*,* Staphylococcus*,* Proteus*,* Klebsiella*,* Pseudomonas*,* E. coli*,* Clostridium perfringens*, and* Vibrio*. The positive effects of* Lactobacillus acidophilus* on the gastrointestinal system are due to adhesion and colonization to the intestinal mucosa, competition for adhesion sites on gut, or other tissue surfaces to prevent pathogens colonization, stimulation of mucosal and systemic immunities, production of antibacterial factors, and special bacteriocin, including acidophilin, lactocidin, acidolin, lactolin, organic acids (lactic acid), and the reduction of PH [10–15]. Probiotics have been used for many purposes, but they are most extensively studied in connection with acute infectious diarrhea, but further research in different age groups and various doses of different probiotics is required to evaluate the impact of probiotics on management of infectious dysentery [9, 16, 17]. Prebiotics are dietary fiber which trigger the growth and activate the activity of a limited number of bacteria in the intestinal flora. In addition, prebiotics can increase the effects of probiotics because of their synbiotic relationships. Synbiotics are combinations of probiotics and prebiotics which can synergistically promote the growth of beneficial bacteria or newly added species in the colon [18]. In this study, we investigated the effects of* Lactobacillus* GG (probiotic) plus prebiotic fructooligosaccharides on dysentery in 1-month–5-year-old children.

## 2. Materials and Methods

The study was conducted between October 2011 and October 2012 at Amirkabir Hospital, Arak, Iran, with a catchment area of 1500000 people.

### 2.1. Description of Participants

The inclusion criteria were male and female patients between the ages of 1 and 60 months who presented with acute dysentery to the PICU or Pediatric Emergency of the Amirkabir Hospital. Participants were patients at the same level of economic conditions who had experienced loose stools with mucus or blood and frequency of more than three times a day for less than two weeks, white blood cell (WBC) count ≥ 5/high-power-field (HPF) in the stool exam (SE), positive stool culture of* Shigella* spp. with or without the presence of fever, abdominal pain, dehydration, anorexia, and vomiting. The criteria for exclusion from the study were refusal of consent by parents, malnutrition status, chronic or concurrent diseases (sepsis, meningitis, pneumonia, and toxic colitis), previous diagnosed acute dysentery, acute abdomen condition, usage of other probiotics, usage of antibiotics or antidiarrheal agents within last 3 days, immune deficiency or treatment with immunosuppressive drugs during the last 60 days, failure to isolate* Shigella* spp. or presence of erythrophagocytic trophozoite of* Entameba histolytica* or cyst/trophozoites of* Giardia lamblia* in the microbial analysis of the stool, and usage of drugs that may have effects on gastrointestinal motility and/or digestion and absorption. The patients who required intravenous fluids, after receiving the treatment in the emergency rooms or PICU, were included in the study as patients with severe or medium dehydration status.

### 2.2. Clinical Management

All patients were examined by a pediatrician. The degree of dehydration, stool appearance, stool consistency, stool frequency, weight loss, duration of dysentery, and fever were recorded. All patients in both groups received the same standard routine treatment such as oral and/or intravenous fluid therapy, antibiotic treatment (Ciprofloxacin, 15 mg/kg, twice a day and for 3 days, orally) and nutritional support. And breastfeeding was promoted.

### 2.3. Randomization, Masking Procedure, and Study Design

In a double-blind manner, the patients were randomized and divided into the placebo and synbiotic groups. Randomization sequence was generated by a computer-generated randomization table in blocks of 4. Except for the study coordinator, all investigators and patients remained blinded to the randomization process until the study was completed. Each patient was given a different code. Parents and the patients were not informed about their allocation status (the synbiotic or placebo group). Placebos and synbiotics were provided by a pharmacist in packages with the same form and were labeled with the code letter A or B. In the production of placebo tablets, the preservative substances and artificial colors had not been used. Also, there was no fermented substance in the tablet. The same as the synbiotic tablets, the placebo tablets did not have any taste. In the PICU or hospital emergency room, the researcher, in a direct and double-blinded manner, supervised the patients to take the tablets properly. The patients in the synbiotic group were given 1 tablet/day of synbiotic tablets (Lactol®), containing probiotic material (bacillus coagulant, 150 million spores per serving) and prebiotic material (fructooligosaccharides, 100 mg per serving) for a period of 3–5 days.

### 2.4. Ethical Approval

The protocol has been written based on guidelines for good clinical practice (GCP) for trials on pharmaceutical products. The protocol approval was obtained from the clinical human research and ethical review committee at the Arak University of Medical Sciences, Iran. The purpose of the study, its objectives, potential benefits, risks, and inconveniences, alternative treatment that may be available, and the subject's rights and responsibilities were explained to the parents. After reading the consent form to the parents in presence of a third party, written informed consent (in accordance with the current revision of the Declaration of Helsinki) was obtained from every parent who wanted their children to participate in the study [19].

### 2.5. Data Analysis

At the end of the study, the study coordinator informed the researcher about the content, synbiotics or placebo, of the packages. Statistical analyses were performed using SPSS (version 12.0., Chicago, USA). An independent sample of *t*-test was administered. Mean ± standard deviation, standard error, Chi squared test, and its non-parametric equivalent (Mann-Whitney) were used to analyze the difference between two groups and find the drug efficacy. *P* < 0.05 was considered statistically significant.

## 3. Results

Out of the patients admitted to the pediatric emergency, 200 patients (out of 961 screened patients) were included in the study. The age of participants was between 1 month and 5 years. The patients were divided into two groups in a double-blind manner; 100 patients were assigned to the synbiotic group and 100 to the placebo group. Before the treatment, there was no difference between the groups in terms of age, gender, degree of dehydration, frequency of stools, or initial period of dysentery. The mean and SD of participants ages were 37.267 ± 22.2 months and 36.933 ± 18.467 in the synbiotic and placebo groups, respectively (*P* > 0.05). In the synbiotic group, there were 54 females (54%) and 46 males (46%). In the placebo group, there were 62 females and 38 males (62% and 38%, resp.; *P* = 0.25).  Table 1 shows the baseline characteristic information related to both synbiotic and placebo groups. Based on these results, age and sex distributions in both synbiotic and placebo groups were similar and no difference was observed among them. Therefore, it can be said that general characteristics of participants do not have any negative influence on the obtained results of the study.

In this study, a comparison between various levels of dehydration shows that, in both synbiotic and placebo groups, a small portion of patients were affected by acute dehydration. In synbiotic group, 84 participants (84%) were affected by minor dehydration, 15 participants (15%) were affected by medium dehydration, and 1 participant (1%) was affected by severe dehydration. In placebo group, 71 participants (71%) were affected by minor dehydration, 25 participants (25%) were affected by medium dehydration, and 4 participants (4%) were affected by severe dehydration (Table 1). The results show that there is no significant relationship between dehydration mean in synbiotic and placebo groups at the beginning of the study (*P* > 0.05). The results obtained by Kolmogorov-Smirnov test show that data distribution is normal. So, the use of independent sample *t*-test is permissible. The demographic findings, mean and standard deviation of duration of dysentery, duration of fever, duration of hospitalization, and the amount of weight loss following the intervention are summarized in Table 2. The mean duration of dysentery was significantly reduced in the synbiotic group when compared to the placebo group (2.5 ± 0.98 days versus 2.9 ± 1.09 days, resp.). Duration of fever after starting treatment was reduced significantly (*P* < 0.001) in children receiving synbiotics (1.64 ± 0.87 days) compared with those in the placebo group (2.13 ± 0.94 days). There was no statistical difference between groups in the mean of hospitalization (3.6 ± 1.04 in synbiotic group versus 3.7 ± 1.08 in placebo group; *P* > 0.05). At the end of the study, Patients taking synbiotics were less likely to have weight loss (129.5 ± 23.3 grams in the synbiotic group versus 278 ± 28.3 grams in the placebo group). There was no death or severe clinical complications during the course of the trial and no adverse effects related to synbiotics were observed.

## 4. Discussion

The present study confirmed the positive effects of probiotics and prebiotics on the treatment of children affected by dysentery. The results of this study showed that routine treatment of dysentery in combination with three to five days of synbiotics reduced the duration of dysentery, duration of fever, and weight changes in children aged between 1 and 60 months. Treatment of acute infectious dysentery is mainly designed to compensate for the dehydration and the loss of electrolytes [20] and to protect the normal gastrointestinal microenvironment [21, 22].

Probiotics are used for this purpose to retrieve the deteriorated normal intestinal microflora. The most investigated probiotics in this field are* Lactobacilli* and* Saccharomyces boulardii* [9]. In spite of the fact that there are numerous studies about probiotics as a treatment for infectious diarrhea, there are some unsolved problems related to the dysentery description, remission criteria, probiotic type, probiotic potent dose, study quality, and probiotic effectiveness evaluation [23]. Recent studies using different probiotics have shown variable effects and meta-analyses show uncertain results due to inequality of studies [24–26]. This suggests that each probiotic has its unique efficacy, so each probiotic needs to be tested to assess its efficacy in specific conditions [27].

Recent systematic reviews recommend further studies of probiotics in an outpatient setting [24, 28]. There are some meta-analyses which have assessed the results of probiotics in the treatment of AGE. In a recent meta-analysis, in order to evaluate the efficacy of probiotics in the treatment of AGE, data was collected from 63 randomized controlled trials [RCTs] and 8014 subjects. 56 out of all those RCTs were carried out in infants and young children. Forty-six RCTs assessed a single probiotic, and 17 RCTs tested a combination of different probiotics.* Lactobacillus* GG,* S. boulardii*, and* Enterococcus* lactic acid bacteria strain SF68 were the most common probiotics used in studies. The Cochrane Review suggested that* Lactobacillus* GG can reduce the duration of diarrhea about 27 hours, stool frequency on the second day, and the probability of diarrhea lasting 4 days. The authors have suggested that more assessments are needed to help clinicians in the use of particular probiotic regimens in specific patient groups [29].

According to a research on the impact of* Lactobacillus reuteri* DSM 17938 on acute infectious diarrhea in a pediatric outpatient setting, it was shown that probiotics had a positive impact on the length of hospitalization and diarrhea. The positive impact on the length of diarrhea was consistent with the findings of the present study [30]. In another study, Golam H. Rabbani et al. studied the impact of green banana on clinical severity of childhood shigellosis. They found that cooked green banana had a positive impact on the length of hospitalization among all age groups. The impact on diarrhea reduction in treatment group was consistent with the findings of the present study [31]. There was no data of weight changes of participants and the impact on the duration of fever was not consistent with the results obtained in the present study.

Ashraf et al. conducted a clinical trial in 2001. In that study, children with confirmed shigellosis were given hyperimmune bovine colostrums in addition to receiving routine treatment. The impact of hyperimmune bovine colostrum on the duration of fever, duration of anorexia, duration of abdominal pain, duration of tenesmus, duration of diarrhea after inclusion, duration of blood in stool, stool frequency on day 3, stool frequency on day 5, cumulative stool frequency in 5-day therapy, number of positive stool cultures on day 3, and number of positive stool cultures on day 5 was investigated in that study. Values were not significantly different between groups. They concluded that HBC as an adjuvant is unable to show any beneficial effect in reducing the severity of childhood shigellosis [32].

In another recent study conducted by Işlek et al. (2014) in Turkey, the role of* Bifidobacterium lactis* B94 plus Inulin in the treatment of acute infectious diarrhea in children was investigated. Compared to the control group, the duration of diarrhea was significantly reduced in the synbiotic group in comparison to the placebo group (3.9 ± 1.2 days versus 5.2 ± 1.3 days, resp.; *P* < 0.001). These results are consistent with the findings of this study in which the positive impact of probiotics/prebiotics on the shortening of duration of diarrhea was observed (2.5 ± 0.98 in synbiotic group and 2.9 ± 1.09 in placebo group). In Işlek et al.'s study, the durations of fever were similar in interference and control groups which were not consistent with the findings of the present study [23].

Vinh et al. conducted a study in 2009. In that study, the impact of Gatifloxacin on the children affected by shigellosis was compared to that of Ciprofloxacin. The results of the study showed that the duration of symptoms in the group receiving Ciprofloxacin was almost the same as that in the group receiving Ciprofloxacin (*P* > 0.05) [33]. They concluded that Ciprofloxacin and Gatifloxacin are similarly effective for the treatment of acute shigellosis. This was not consistent with the findings of the present study.

There is a randomized double-blinded, placebo-controlled clinical trial study performed by Chen et al. (2010) on 304 children aged 3 months to 6 years with acute infectious diarrhea. The patients received Bio-Three (a mixture of* Bacillus mesentericus*,* Enterococcus faecalis*, and* Clostridium butyricum*) or placebo for one week in Chang Gung Children's Hospital in Northern Taiwan. In comparison to the placebo group, the Bio-Three group presented a significant reduction in the severity and duration of diarrhea and the duration of hospital stay, although no reduction of the duration of fever was observed [34]. The Working Group on Probiotics of the European Society for Pediatric Gastroenterology, Hepatology and Nutrition (ESPGHAN) described that the use of probiotics should be considered as an adjuvant therapy to ORS in the management of acute gastroenteritis [9, 24].

In the literature, the properties of probiotics have been recognized as a safe and beneficial adjunct to many treatments for infections [35–38]. In our study, no adverse effect toward synbiotics has been reported as well; but recently it has been a matter of intense debate so that in a review article it has been concluded that since there may be different strains of different probiotics with different properties it is possible to have different results of efficacy or adverse effects [39].

## 5. Conclusion

Dysentery is one of the most common diseases among children. This disease has harmful impacts on children, family, and society. Due to harmful consequences in terms of economy, human loss, and also the lack of definite treatment which leads to the resistant form of the disease, using probiotics can be beneficial. The findings of this study indicate the beneficial effects of* Lactobacillus* as an adjunct to standard treatment on the children affected by dysentery, shortening duration of fever and duration of dysentery. Besides, many studies have confirmed the lack of side effects of probiotics. Therefore, it seems that using probiotics/prebiotics as a tool of side treatment in areas affected by dysentery can be beneficial to improve children's health.

## Figures and Tables

**Table 1:** General characteristics of patients in both synbiotic and placebo groups.

Characteristic	Synbiotic	Placebo	*P* value
Age, mean ± SD, months	37.267 ± 22.2	36.933 ± 18.467	0.9
Sex (male/female)	46%/54%	38%/62%	0.25
Dehydration			
Minor	84%	71%	0.067
Medium	15%	25%	
Severe	1%	4%	

**Table 2:** Mean and standard deviation of patient characteristics during the study.

Characteristics	Synbiotic	Placebo	*P* value
Duration of dysentery (day)	2.5 ± 0.98	2.9 ± 1.09	0.01
Duration of fever (day)	1.64 ± 0.87	2.13 ± 0.94	<0.001
Duration of hospitalization (day)	3.6 ± 1.04	3.7 ± 1.08	0.691
Weight loss (gram)	129.5 ± 23.3	278 ± 28.3	<0.001
